# Pharmacokinetic Study of Carboplatin Using Various Overweight-Correcting Dosing Algorithms and Biomarkers in Patients With Varying BMI Categories

**DOI:** 10.1097/FTD.0000000000001328

**Published:** 2025-04-29

**Authors:** Mart P. Kicken, Corine Bethlehem, Karin Beunen, Peter de Jong, Theo van Voorthuizen, Jeanine J. van den Hudding, Dirk-Jan A.R. Moes, Matthijs van Luin, Rob ter Heine, Hans J.M. Smit, P. Margreet G. Filius, Maarten J. Deenen

**Affiliations:** * Department of Clinical Pharmacy, Rijnstate Hospital, Arnhem, the Netherlands;; † Department of Clinical Pharmacy, Catharina Hospital, Eindhoven, the Netherlands;; ‡ Department of Pharmacy, Radboud University Medical Center, Radboud Institute for Health Sciences, Nijmegen, the Netherlands;; § Department of Clinical Pharmacy, Erasmus University Medical Center, Rotterdam, the Netherlands;; ¶ Department of Internal Medicine, Rijnstate Hospital, Arnhem, the Netherlands;; ║ Department of Pulmonology, Rijnstate Hospital, Arnhem, the Netherlands;; ** Department of Clinical Pharmacy and Toxicology, Leiden University Medical Center, Leiden, the Netherlands; and; †† Department of Clinical Pharmacy, Meander Medical Center, Amersfoort, the Netherlands.

**Keywords:** carboplatin, dose adaptation, body weight, pharmacokinetics, Cockcroft–Gault formula

## Abstract

**Background:**

The Cockcroft–Gault (CG) formula is more likely to overestimate renal function and carboplatin dosing in patients who are overweight. In this prospective pharmacokinetic study, the use of an adjusted Cockcroft–Gault formula (aCG) was evaluated to correct for overweight status.

**Methods:**

aCG was adjusted in patients with body mass index (BMI) >25 kg/m^2^ using adjusted ideal body weight, capping low serum creatinine values at 60 μmol/L, and high creatinine clearance values at 125 mL/min. Patients were categorized as follows: BMI <25.0 (normal weight), 25.0–29.9 (overweight), and ≥30.0 kg/m^2^ (obese). To assess the pharmacokinetics, blood samples were collected and carboplatin ultrafiltrate concentrations were analyzed. Exposure was estimated using a population pharmacokinetic model and compared with the target area under the curve (AUC) regarding bias mean prediction error (MPE%) and imprecision mean absolute prediction error (MAPE%). In addition, substitutes for renal function, including weight descriptors, cystatin C, 24-hour creatinine clearance, and glomerular filtration rate estimators, were compared.

**Results:**

Eighteen patients were included in this study. aCG slightly underestimated individual carboplatin clearance across all weight groups, with the highest deviation in patients who had obesity (MPE%: −10.5%) versus +8.8% using CG. aCG underestimated −5.7% in patients with a normal weight and overestimated by +1.1% in those who were overweight, compared with −4.2% and +2.8%, respectively, using CG. The most accurate predictor of the target AUC for all weight categories was cystatin C (MPE%: +0.2%, −2.0%, and −0.1% for patients with a normal weight, those who were overweight, and those who had obesity, respectively) with low imprecision (MAPE%: 9.8%, 9.5%, and 13.3%, respectively).

**Conclusions:**

This study did not find evidence to support using aCG to predict carboplatin clearance better than CG. However, cystatin C was found to be the most precise and accurate biomarker for carboplatin clearance.

## Abstract 2

Supplemental Digital Content is Available in the Text.

## INTRODUCTION

Carboplatin is mainly excreted by the kidneys, where up to 50%–75% of the total platinum is excreted within 24 hours after administration.^1–3^ Carboplatin dosing is adjusted for renal function, as it is linearly correlated with the glomerular filtration rate (GFR).^4–7^ In addition, several studies have found an association between systemic carboplatin exposure and efficacy and toxicity. Hence, carboplatin dosing is based on a targeted systemic carboplatin exposure expressed as the area under the concentration–time curve (AUC).^1,8–13^ The target AUC values typically range between 4 and 7 mg*min/mL and depend on the type of treatment regimen and dose interval frequency.^4^

Several formulas exist for calculating individual carboplatin dosages. The Calvert formula is the most widely used worldwide.^1,2,4,5,14–16^ In this formula, the GFR is generally substituted with the estimated creatinine clearance (CrCL) using the Cockcroft–Gault (CG) formula.^14^ For different formulas, see **Supplemental Digital Content 1** (http://links.lww.com/TDM/A834). The CG formula is well suited for estimating CrCL in patients with normal weight and normal serum creatinine levels. However, as creatinine is primarily produced by skeletal muscle (ie, muscle mass), the CG estimation of creatinine clearance is not directly biased by body weight itself but rather by discrepancies between absolute body weight and lean body mass. Hence, the estimated CrCL is more likely to be overestimated in patients who are overweight (as an increase in absolute body weight does not necessarily correspond to a proportional increase in muscle mass) and patients with low serum creatinine values, who are typically cachectic patients. Indeed, multiple studies have shown that a high body mass index (BMI) or low serum creatinine level is independently associated with the overestimation of creatinine clearance, thereby increasing the risk of carboplatin toxicity.^10,11,15–21^

**FIGURE 1.**
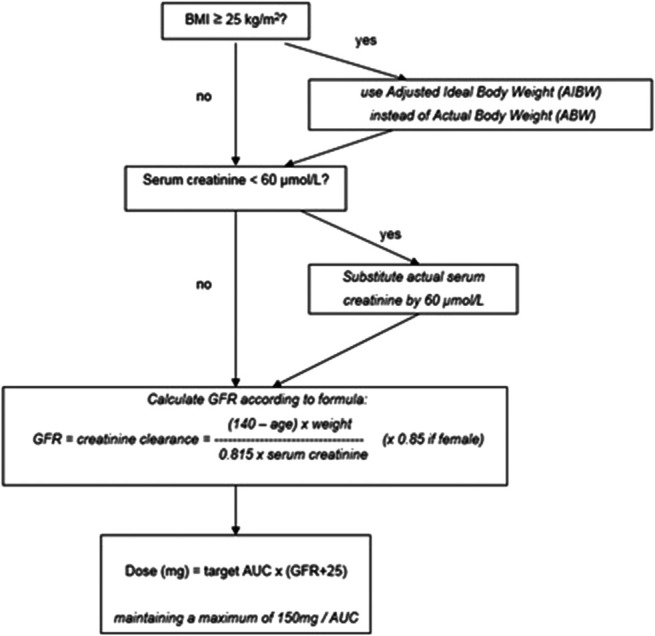
Proposed adjusted dosing algorithm of carboplatin. aCG, adjusted Cockcroft-Gault; BMI, body mass index; GFR, glomerular filtration rate; AUC, area under the curve.

We hypothesized that the potential overestimation and increased risk of carboplatin-associated severe toxicity could be prevented by adjusting for high BMI and low serum creatinine levels. Hence, we designed an adjusted Cockcroft–Gault (aCG) dosing algorithm based on available evidence and guidelines regarding carboplatin dosing in patients who were overweight and those with cachexia (Fig. 1), with the ultimate aim of improving the safe dosing of carboplatin in these patients. The aCG adjusted for overweight by using adjusted ideal body weight (AIBW) instead of absolute body weight (ABW) in patients with BMI ≥25 kg/m^2^ and for cachexia by substituting serum creatinine values < 60 μmol/L with 60 μmol/L.^2,22,23^ Indeed, studies have shown that using AIBW instead of actual body weight better approximates the target AUC in patients who are overweight or have obesity.^2,22,24,25^ Moreover, multiple guidelines proposed using an alternative weight descriptor such as AIBW in patients with a BMI≥25 kg/m^2^ and affirm the minimum cut-off value of 60 μmol/L for serum creatinine.^26,27^ Finally, we adjust for overestimating CrCL by capping the maximum estimated CrCL at 125 mL/min^26–29^

In this prospective pharmacokinetic study, we evaluated the performance of an adjusted dosing algorithm in which the measured carboplatin AUCs were compared with the target AUCs. The secondary objective was to investigate and compare the performance of aCG with other substitutes for renal function based on measured carboplatin exposure, including other weight descriptors, cystatin C, 24-hour creatinine clearance, and estimated GFR by the Chronic Kidney Disease Epidemiology Collaboration (CKD-EPI).

## MATERIALS AND METHODS

### Study Design

We conducted a prospective study at Rijnstate Hospital in Arnhem, Netherlands. The primary objective was to evaluate the pharmacokinetics and safety of our adjusted carboplatin dosing algorithm that adjusted for overweight, defined as BMI≥ 25 kg/m^2^, low serum creatinine, defined as serum creatinine <60 μmol/L, and maximal estimated creatinine clearance, defined as a maximum estimated CrCL of 125 mL/min (Fig. 1). The secondary objective was to investigate and compare the performance of aCG with other substitutes for renal function based on the measured carboplatin clearance, including other weight descriptors, cystatin C, using the formula of Schmitt et al,^16^ 24-hour creatinine clearance, and CKD-EPI estimating the GFR (eGFR). The administered dose of carboplatin was calculated using aCG. Blood samples were collected for pharmacokinetic measurements on day 1 of the first treatment cycle. Further treatment was provided according to the standard treatment protocols and routine clinical care. This study was approved by the local medical ethics committee and followed the principles of the Declaration of Helsinki. Written informed consent was obtained from all the patients.

### Patient Population

Patients were included if they were aged 18 years or older; had a histologically or cytologically proven non-small-cell lung carcinoma (NSCLC), small-cell lung carcinoma, ovarian or endometrial cancer for which they were treated with carboplatin at a target AUC of 4, 5, or 6 mg*min/mL; had an estimated life expectancy of at least 12 weeks; had a WHO performance status of 0–2. Furthermore, patients had to have adequate baseline liver function and bone marrow defined as hemoglobin ≥6.0 mmol/L, white blood cell count ≥3.0 × 10^9^/L, absolute neutrophil count (ANC) ≥1.5 × 10^9^/L, platelet count ≥100 × 10^9^/L, bilirubin ≤1.5 times the upper limit of normal (ULN), and ALAT and ASAT ≤2.5 times ULN (in case of liver metastases ≤5.0 times ULN). Patients that were included were categorized into three different BMI categories: <25.0, 25.0–29.9, or ≥30.0 kg/m^2^. Patients were excluded if they were treated with carboplatin at a target AUC below 4 mg*min/mL; had an active clinically serious infection or a history of a kidney allograft; were pregnant or breastfeeding; and were unsuitable for follow-up. This study was completed 4 weeks after the last participant received their last cycle of carboplatin.

### Blood and Material Sampling

The pharmacokinetics of carboplatin were determined by obtaining 5 blood samples in 4 mL heparinized collection tubes on day 1 of the first cycle of treatment: 1 sample was taken before the start of the carboplatin infusion, 1 sample at the end of the infusion (t = 0), and 1 at t = 1, 2.5, and 5 hours after the end of the infusion. In addition, a 24-hour creatinine clearance and cystatin C samples were collected from the patients the day before the start of treatment to estimate renal clearance. Immediately after blood drawing to obtain plasma, the blood samples were centrifuged at 4°C at 1500*g* for 10 minutes. Next, the plasma ultrafiltrate was obtained by centrifuging 1 mL of the plasma for 15 minutes using an ultrafiltrate filter (Merck Millipore, Tullagreen, Cork, Ireland). The plasma ultrafiltrate was stored at −70°C until analysis.

### Bioanalysis

The concentrations of carboplatin in the human plasma ultrafiltrate were determined using a validated graphite-furnace atomic absorption spectrometry assay with slight modifications in the optimization settings as previously described.^30,31^

### Pharmacokinetic Analysis

The AUCs of patients' ultra-filterable carboplatin concentrations, when dosed according to aCG, were estimated using a post hoc estimation in NONMEM (FOCE + I) using the 2-compartment nonlinear mixed-effects model for carboplatin described by Ekhart et al.^2^ All estimations and simulations were performed using the nonlinear mixed-effects modeling software package NONMEM V7.4.4 (Development Solutions, Ellicott City, MD). Perl Speaks NONMEM (v.5.0.0). Pirana (v.2.9.8) and R statistics (v.4.2.3) were used for interpretation and visualization. Next, we accounted for dose rounding when estimating AUC. For example, if the calculated carboplatin dosage at the target AUC of 5 for a typical patient was 620 mg, the actual dosage administered would have been 600 mg owing to dose rounding.

Next, different weight descriptors for estimating creatinine clearance were converted into an estimated AUC by dividing the administered dose by the calculated carboplatin clearance (estimated CrCL+25 based on Calvert^4^). See **Supplemental Digital Content 1 (**http://links.lww.com/TDM/A834) for all carboplatin dosing formulas. For example, the conventional CG formula using ABW provides an estimated CrCL of 80 mL/min for patients receiving 600 mg, resulting in an estimated AUC of 5.7 mg*min/mL. In this way, the estimated AUCs of different weight descriptors ABW and AIBW using the conventional CG formula were estimated, either with or without substituting serum creatinine values < 60 μmol/L with 60 μmol/L and capping estimated CrCL at 125 mL/min. Moreover, the weight descriptors of Bénézet et al^32^ using the ideal body weight (IBW) and absolute body weight were compared. For an overview of the equations of different weight descriptors, see **Supplemental Digital Content 2** (http://links.lww.com/TDM/A834).

Finally, the AUC was estimated for 24-hour creatinine clearance; for cystatin C, the formula by Schmitt et al^16^; for flat dosing based on the mean carboplatin population clearance^2,15^; and for the GFR estimators: the 2021 CKD-EPI creatinine and CKD-EPI creatinine-cystatin C equations.^33^

### Sample Size Calculation and Statistical Analysis

A total of 7 patients per BMI category (<25.0, 25.0–29.9, and ≥30.0 kg/m^2^) were required to detect an anticipated difference of 30% between the actual versus the target AUC of carboplatin, with a SD of 1.2 mg/mL*min, an alpha of 0.05%, and a power of 80%. We anticipated based on previous data that at least 1 in 4 patients would have a serum creatinine concentration below 60 µmol/L. Data were analyzed per protocol analysis. Patient characteristics and pharmacokinetic data were analyzed using descriptive statistics. Continuous variables were analyzed using the Kruskal–Wallis test. All statistical tests were performed using IBM SPSS Statistics for Windows, Version 25.0 (IBM, Armonk, NY).

### Study End Points

The primary end points of this study were the mean prediction error (MPE%) and mean absolute prediction error (MAPE%) of the aCG algorithm, which were expressed using the measured plasma concentration–time curve (actual AUC) of the carboplatin ultrafiltrate. The acceptance criteria for the MPE% and MAPE% were within 85% and 115% of the actual and estimated AUC, respectively, versus the target AUC.

The secondary end points were the MPE% and MAPE% of other substitutes for renal function based on the measured clearance, including other weight descriptors (Bénézet et al,^32^ AIBW in CG), capping CG, cystatin C-based estimates using the Schmitt et al^16^ formula (which besides cystatin C also incorporates additional patient characteristics), 24-hour creatinine clearance, and eGFR formulas of the CKD-EPI creatinine and CKD-EPI creatinine-cystatin C.^33^

Safety parameters included the incidence of hematological and nonhematological toxicity, toxicity-related hospitalization, carboplatin dosage reduction, and treatment delay. Toxicity was assessed using the Common Terminology Criteria for adverse events (CTCAE) v5.0.^34^

## RESULTS

### Study Population

A total of 21 patients were included in this study. General patient and treatment characteristics are shown in Table 1. Pharmacokinetic sampling was completed successfully in 18 of the 21 included patients and failed in 2 patients (plasma was collected instead of ultrafiltrate); for 1 patient, only 1 blood sample was collected. There were 7 patients with a BMI <25.0 kg/m^2^, 5 with 25.0–29.9 kg/m^2^, and 6 with ≥30.0 kg/m^2^.

**TABLE 1.** Population Characteristics of Different BMI Subgroups

Characteristics	Normal and Underweight (BMI <25.0 kg/m^2^)	Overweight (BMI 25.0 to 29.9 kg/m^2^)	Obese (BMI ≥30.0 kg/m^2^)
N (%)	7 (37)	5 (32)	6 (32)
Age [yr], median (range)	68 (50–78)	60 (54–77)	66 (56–78)
Sex, n (%)			
Male	6 (86)	1 (20)	3 (50)
Female	1 (14)	4 (80)	3 (50)
Weight [kg], median (range)	68 (48–80)	76 (72–81)	99 (87–115)
Length [cm], median (range)	179 (171–185)	170 (166–173)	170 (160–180)
BSA [m^2^], median (range)	1.8 (1.5–2.0)	1.9 (1.8–2.0)	2.2 (2.0–2.4)
Baseline serum creatinine [µmol/L], median (range)	69 (49–95)	82 (67–130)	77 (57–114)
24 hours creatinine [mmol/L/24 hours], median (range)	9.8 (3.8–11.8)	8.6 (7.7–10.5)	14.5 (7.7–16.3)
24 hours creatinine clearance [mL/min/24 hours], median (range)	105.1 (98.4–110.4)	69.6 (46.5–108.5)	122.8 (71.6–139.4)
Cystatin C [mg/L], median (range)	0.9 (0.8–1.6)	1.5 (0.8–1.9)	1.3 (0.8–1.5)
Target AUC [mg*min/mL], median (range)	6.0 (6.0–6.0)	6.0 (5.0–6.0)	6.0 (5.0–6.0)
Primary tumor, n (%)			
NSCLC	6 (86)	1 (20)	3 (50)
SCLC	1 (14)	3 (60)	2(33)
Other	0 (0)	1 (20)	1 (17)
Concurrent therapies, n (%)			
Paclitaxel	4 (57)	2 (40)	2 (33)
Gemcitabine	2 (29)	0 (0)	1 (17)
Pemetrexed	0 (0)	0 (0)	1 (17)
Etoposide	1 (14)	3 (60)	4 (67)
Bevacizumab	1 (14)	0 (0)	2 (25)
Radiotherapy	0 (0)	1 (20)	1 (13)

BMI, body mass index; NSCLC, non-small cell lung carcinoma; SCLC, small cell lung carcinoma; AUC, area under the curve; BSA, body surface area.

### Pharmacokinetic Analysis

**FIGURE 2.**
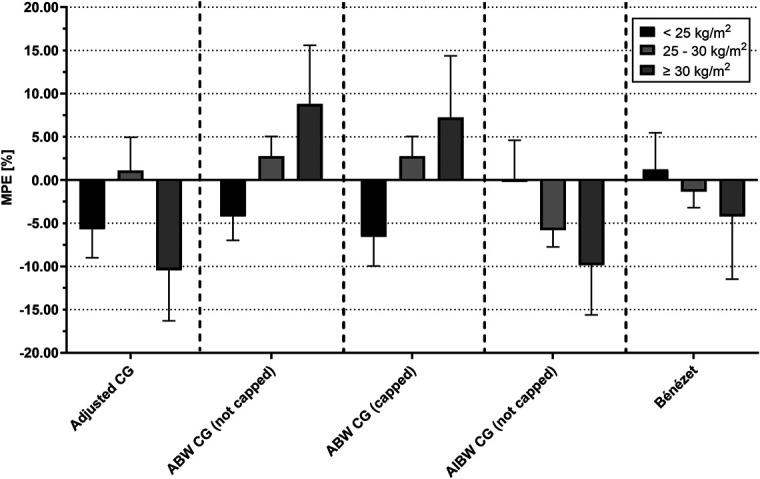
AUC (expressed as MPE%) of different weight descriptors relative to the target AUC. AUC, area under the curve; MPE%, mean percentage error; (a)CG, (adjusted) Cockcroft-Gault; ABW, absolute body weight; AIBW, adjusted ideal body weight. The margin of error is equal to the standard error (SE) of the mean for a particular weight descriptor.

Table 2 lists the MPE% and MAPE% of the estimated AUCs for various weight descriptors. The actual AUC_0-∞_ following dosing according to the aCG formula across all BMI subgroups was slightly lower compared with the target AUC with the highest MPE% deviation of −10.5% (95% confidence interval [CI], −21.9 to 1.0) in patients with BMI ≥30.0 kg/m^2^ compared with +8.8% (95% CI, −4.5 to 22.1) when the conventional CG was used. Patients with a BMI <25.0 kg/m^2^ or 25.0–29.9 kg/m^2^ had an underestimation of −5.7% and +1.1% using aCG, while the conventional CG formula gave an under- and overestimation of −4.2% and +2.8%, respectively (Fig. 2). Furthermore, a higher BMI was associated with an increase of MPE% for conventional CG, either uncapped or capped, ranging from an underestimation of −6.6% in BMI <25.0 kg/m^2^ to an overestimation of +8.8% in ≥30.0 kg/m^2^. See **Supplemental Digital Content 3 (**http://links.lww.com/TDM/A834) for the concentration curves and exposure of each patient.

**TABLE 2.** Primary Outcomes of Different Weight Descriptors and Estimators of GFR

	BMI <25.0 kg/m^2^ (n = 7)			BMI 25.0 to 29.9 kg/m^2^ (n = 5)			BMI ≥30.0 kg/m^2^ (n = 6)		
	AUC [mg*min/mL]	MPE% [95% CI]	MAPE% [95% CI]	AUC [mg*min/mL]	MPE% [95% CI]	MAPE% [95% CI]	AUC [mg*min/mL]	MPE% [95% CI]	MAPE% [95% CI]
Target AUC (reference)	5.9 (5.7–6.1)			5.8 (5.4–6.1)			5.7 (5.4–6.1)		
Adjusted Cockcroft–Gault	5.6 (5.2–6.0)	−5.7 (−12.2 to 0.8)	7.9 (3.0–12.7)	5.7 (5.6–5.9)	1.1 (−6.4 to 8.6)	5.4 (0.0–10.9)	5.1 (4.4–5.8)	−10.5 (−21.9 to 1.0)	13.6 (5.0–22.2)
Weight descriptors									
Conventional Cockcroft–Gault									
ABW (not capped)	5.7 (5.4–6.0)	−4.2 (−9.6 to 1.1)	7.1 (4.3–10.0)	5.9 (5.3–6.4)	2.8 (−1.7 to 7.2)	4.5 (1.7–7.3)	6.2 (5.4–7.0)	8.8 (−4.5 to 22.1)	15.7 (8.7–22.6)
ABW (capped)*	5.5 (5.2–5.9)	−6.6 (−13.2 to 0.1)	8.7 (3.9–13.5)	5.9 (5.3–6.4)	2.8 (−1.7 to 7.2)	4.5 (1.7–7.3)	6.1 (5.3–7.0)	7.2 (−6.7 to 21.2)	15.5 (8.3–22.7)
AIBW (not capped)	5.9 (5.4–6.4)	0.1 (−8.8 to 9.0)	10.7 (8.4–13.0)	5.4 (4.9–5.9)	−5.8 (−9.6 to (−2.0)	5.8 (2.0–9.6)	5.2 (4.5–5.8)	−9.9 (−21.1 to 1.4)	13.0 (4.5–21.5)
Bénézet equation^32^	6.0 (5.5–6.5)	1.2 (−7.1 to 9.5)	9.8 (6.9–12.8)	5.8 (5.5–6.1)	−1.3 (−5.0 to 2.3)	2.5 (−0.3 to 5.2)	5.6 (4.9–6.3)	−4.2 (−18.4 to 10.0)	12.8 (4.9–20.7)
Estimators of GFR									
24-hour creatinine clearance†	6.0 (4.9–7.0)	2.2 (−16.3 to 20.7)	17.7 (7.6–27.9)	6.0 (5.1–7.0)	7.0 (−4.5 to 18.6)	8.6 (−1.6 to 18.7)	4.7 (3.1–6.3)	−17.6 (−45.8 to 10.6)	19.7 (−7.1 to 46.5)
Cystatin C‡^,^^16^	5.9 (5.4–6.4)	0.2 (−8.9 to 9.2)	9.8 (5.1–14.4)	5.8 (5.2–6.3)	−2.0 (−12.9 to 8.8)	9.5 (6.4–12.5)	5.8 (5.0–6.7)	−0.1 (−17.7 to 17.5)	13.3 (1.5–25.1)
CKD-EPI (creatinine)^33^	6.3 (5.7–6.7)	5.5 (−3.6 to 14.7)	11.1 (6.1–16.1)	5.9 (5.5–6.3)	−0.2 (−8.4 to 8.1)	6.5 (2.9–10.2)	5.3 (4.4–6.2)	−9.5 (−26.4 to 7.3)	15.2 (3.1–27.4)
CKD-EPI (creatinine-cystatin C)^33^	5.9 (5.2–6.7)	0.4 (−13.3 to 14.1)	14.9 (8.2–21.6)	5.5 (4.5–6.4)	−7.0 (−24.2 to 10.2)	14.4 (4.9–24.0)	4.8 (4.0–5.6)	−17.8 (−33.1 to (−2.4))	21.0 (−33.1 to 31.7)
Flat dosing§^,^^2,15^	5.8 (5.3–6.4)	−1.6 (−10.2 to 6.9)	8.6 (3.4–13.8)	7.1 (5.9–8.3)	23.9 (7.1–40.7)	23.9 (7.1–40.7)	5.6 (4.1–7.1)	−2.2 (−28.6 to 24.2)	26.3 (13.4–39.3)

* Estimated creatinine clearance capped at the upper limit of 125 mL/min, serum creatinine capped at the lower limit of 60 µmol/L.

† Four patients were excluded for having insufficient measurements to determine 24-hour creatinine clearance.

‡ Two patients were excluded for having insufficient measurements.

§ Based on the mean carboplatin population clearance of 112.4 mL/min. GFR, glomerular filtration rate; BMI, body mass index; AUC, area under the curve; MPE%, mean percentage error; MAPE%, mean absolute percentage error; 95% CI, 95% confidence interval; ABW, absolute body weight; AIBW, adjusted ideal body weight; GFR, glomerular filtration rate; CKD-EPI, Chronic Kidney Disease Epidemiology Collaboration.

**FIGURE 3.**
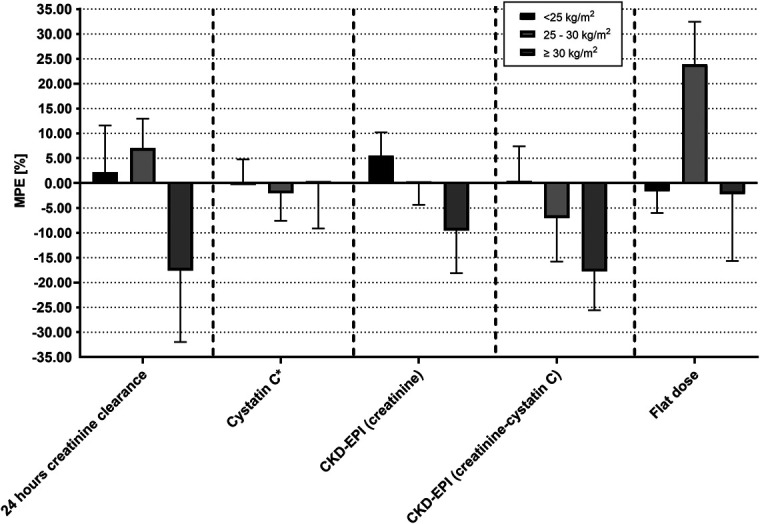
AUC (expressed as MPE%) of different estimators of GFR relative to target AUC.

The most accurate predictor of target AUC across all BMI categories was cystatin C, with a minimal deviation in MPE% between −2.0% and +0.2%, followed by the Bénézet equation with +1.2% for BMI <25.0 kg/m^2^, −1.3% for BMI 25.0–29.9 kg/m^2^, and −4.2% for BMI ≥30.0 kg/m^2^. All MPE% remained between 85% and 115% of the actual AUC irrespective of weight descriptor or formula for renal function used, except for 24-hour creatinine clearance [MPE% = −17.6% (95% CI, −45.8 to 10.6)] and CKD-EPI (creatinine-cystatin C) in patients with BMI ≥30.0 kg/m^2^ [MPE% = −17.8% (95% CI, −33.1 to −2.4)], and flat dose in patients with BMI 25.0–29.9 kg/m^2^ [MPE% = 23.9% (95% CI, 7.1–40.7)] (Table 2). All MAPE% ranged between 2.5% and 26.3% and were the highest for flat dose, 24-hour creatinine clearance, and CKD-EPI (creatinine and creatinine-cystatin C), and in the BMI ≥30.0 kg/m^2^ group compared with the other BMI groups Figure 3).

The mean clearance rate in the carboplatin population clearance was 112.4 mL/min. Flat dosing resulted in an overestimation of +23.9% (95% CI, 7.1–40.7) for BMI 25.0–29.9 kg/m^2^ and an underestimation of −1.6% (95% CI, −10.2 to 6.9) for <25.0 kg/m^2^ and −2.2% (95% CI, −28.6 to 24.2) for ≥30.0 kg/m^2^. CKD-EPI (creatinine) showed a 5.5% overestimation in patients with BMI <25.0 kg/m^2^, near perfect approximation for patients with BMI 25.0–29.9 kg/m^2^ (MPE% = −0.2%) and −9.5% underestimation in patients with BMI ≥30.0 kg/m^2^. The same trend was seen for CKD-EPI (creatinine-cystatin C) with respectively overestimation of 0.4% in patients with BMI <25.0 kg/m^2^, and underestimation in 25.0–29.9 kg/m^2^ −7.0% and −17.8% in ≥30.0 kg/m^2^ (see **Supplemental Digital Content 4**, http://links.lww.com/TDM/A834).

### Treatment and Toxicity Outcomes

The patients were treated with a median of 3 cycles. Table 3 shows the treatment and toxicity outcomes of dosing according to aCG across different BMI subgroups.

**TABLE 3.** Patient Treatment Characteristics of Different BMI Subgroups

Parameter	General (n = 18)	Normal and Underweight (BMI <25.0 kg/m^2^) (n = 7)	Overweight (BMI 25.0–29.9 kg/m^2^) (n = 5)	Obese (BMI ≥30.0 kg/m^2^) (n = 6)
End of treatment, n (%)	9 (50)	2 (29)	4 (80)	3 (50)
Number of cycles of treatment planned, median (range)	4 (3–6)	4 (4–5)	4 (3–6)	4 (4–6)
Number of cycles of treatment performed, median (range)	3 (1–6)	2 (1–5)	4 (3–6)	3 (1–5)
Hematological toxicity (any CTCAE grade), n (%)	15 (83%)	5* (83%)	5 (100%)	5 (83%)
Nonhematological toxicity, n (%)	4 (22%)	2 (29%)	0 (0%)	1 (17%)
Toxicity-related hospitalization, n (%)	4 (22%)	1 (14%)	1 (20%)	1 (17%)
Toxicity related to dosage reduction, n (%)	1 (6%)	0 (0%)	0 (0%)	1 (17%)
Toxicity-related delay treatment, n (%)	6 (33%)	2 (29%)	3 (60%)	1 (17%)
Delay treatment (d), median (range)	0 (0–14)	0 (0–7)	6 (0–14)	0 (0–8)
Hematological toxicity				
Hemoglobin				
CTCAE grades 1–2, n (%)	12 (71%)	3*	4	5
CTCAE grades 3–4, n (%)	4 (24%)	3*	1	0
Leukocytes				
CTCAE grades 1–2, n (%)	7 (42%)	3*	3	1
CTCAE grades 3–4, n (%)	4 (24%)	0*	2	2
Thrombocytes				
CTCAE grades 1–2, n (%)	2 (12%)	1*	1	0
CTCAE grades 3–4, n (%)	4 (24%)	1*	2	1
Neutrophils†				
CTCAE grades 1–2, n (%)	2 (33%)	1	1	0
CTCAE grades 3–4, n (%)	2 (33%)	0	0	2

* One patient had no hemoglobin, leukocyte, or thrombocyte measurements.

† Only 6 patients (respectively 1, 2, and 3 patients) had their neutrophils determined.

BMI, body mass index; CTCAE, Common Terminology Criteria for Adverse Events version 5.0.^34^

## DISCUSSION

We conducted a pharmacokinetic study to prospectively investigate the performance of an alternative dosing algorithm (aCG) for carboplatin in patients in different BMI categories. Our study did not confirm that administration based on aCG resulted in improved exposure compared with the conventional CG formula. In addition, other formulae previously describing the pharmacokinetics of carboplatin were assessed for performance, of which cystatin C (based on the formula of Schmitt et al^16^) provided the best approximation of the target AUC, independent of weight, expressed as BMI.

Although our aCG did not approximate the target AUC better than the conventional CG, it showed slight underdosing in patients who were overweight (BMI ≥30 kg/m^2^). The underestimation of carboplatin dosing in patients who were overweight could be explained by the increased bias in using the IBW to calculate the AIBW. A similar underestimation trend was observed in our study for the weight descriptor of Bénézet. AIBW, instead of absolute body weight, considers muscle mass more accurately after adjusting for sex and fat mass. The possible use of AIBW instead of ABW is in line with other studies that show that AIBW better predicts the target AUC in patients who are overweight and obese, whereas ABW overestimates the carboplatin AUC.^2,22,24,25^ In addition to body weight adjustment using AIBW, another adjustment in our formula was capping serum creatinine at 60 µmol/L, which is especially important in patients with sarcopenic obesity (loss of muscle mass combined with increased fat mass).^35^ Indeed, multiple studies have shown a benefit for capping low creatinine values, typically seen in patients with cachexia.^22,23^ Guidelines of the Gynecologic Oncology Group and the National Comprehensive Cancer Network recommend the use of a minimum cut-off of serum creatinine of 0.7 mg/dL (∼60 μmol/L) for all weight classes and capping estimated CrCL at 125 mL/min^26–29^ Moreover, these guidelines recommend using an alternative weight descriptor, such as AIBW, for patients with a BMI ≥25.0 kg/m^2^.^26,27^ In our study, only 2 patients had a serum creatinine below 60 μmol/L. Therefore, it was not possible to reach a conclusion regarding the minimization of serum creatinine and maximization of renal function with carboplatin dosing. Furthermore, only 1 patient in the ≥30 kg/m^2^ group had the estimated CrCL capped to 125 mL/min (from 139.54 mL/min), making it impossible to evaluate to what extent capping estimated creatinine clearance increases the risk of carboplatin underdosing across different weight classes.

Carboplatin's clearance is determined by the GFR. Calvert initially used the 100% glomerular filtered ^51^Cr-EDTA as an ideal predictor of GFR.^4^ Creatinine, however, is not solely cleared by the GFR but also undergoes active secretion by the peritubular capillaries in the kidneys resulting in a 10%–20% overestimation of GFR.^36^ In our study, the different weight descriptors provided an adequate approximation of target exposure in patients who were underweight and those with a normal weight (−5.7% to +2.8%). However, other estimators of GFR and carboplatin clearance performed better in each BMI group, especially the biomarker cystatin C. Cystatin C is a direct biomarker for glomerular filtration rate because it is produced at a constant rate and is 100% freely filtered at the glomerulus, and neither secreted nor reabsorbed at the proximal or distal renal tubule.^37^ In our study, cystatin C provided the best approximation of the target AUC independent of BMI. This aligns with the original Calvert formula that initially used the 100% glomerular filtered ^51^Cr-EDTA as an ideal predictor of GFR.^4^ However, the formula of Schmitt et al^16^ uses body composition in addition to cystatin C and also includes the parameters ABW, age, sex, and serum creatinine. Therefore, it is impossible to identify cystatin C as a sole predictor of carboplatin clearance. Similarly, CKD-EPI (creatinine-cystatin C) uses serum creatinine, cystatin C, sex, and age of the patient.^33^ Nonetheless, in our study, CKD-EPI (creatinine-cystatin C) provided an underestimation of carboplatin exposure dependent on BMI, ranging from +0.4% for patients with a normal weight to −7.0% for those who were overweight and −17.8% for those who had obesity. This may indicate that using only cystatin C, without adjusting for weight, is insufficient for estimating carboplatin exposure. Indeed, multiple prospective studies have shown that cystatin C, in addition to other covariates such as serum creatinine, body weight, age, and sex, can accurately predict carboplatin clearance.^17,38^

Carboplatin dosing based on body weight has been a topic of discussion for some time. A large study by Ekhart et al^15^ in patients with NSCLC receiving carboplatin compared different weight descriptors, including AIBW, IBW, FFM (fat-free mass), and LBM (lean body mass), and the Bénézet equation found flat dosing to be the best weight descriptor in patients with a BMI ≥25.0 kg/m^2^, calling to question whether weight altogether is even correlated with carboplatin exposure. In our study, flat dosing, as with cystatin C, showed perfect estimation of carboplatin exposure in underweight and overweight groups and only provided an overestimation in the BMI 25.0–29.9 kg/m^2^ group (see **Supplemental Digital Content 4**, http://links.lww.com/TDM/A834). This result, together with the results of cystatin C, suggests that weight is not strongly correlated to carboplatin exposure, as already proposed by some other studies.^2,15^ Moreover, a large study (n = 491) by White-Koning et al^39^ comparing different formulas with actual carboplatin clearance found CKD-EPI with cystatin C to be the best predictor of carboplatin clearance, independent of any patient characteristics such as sex, BMI (only significant at the 1% level), age and eGFR. Finally, besides weight not being correlated strongly to carboplatin clearance, hydrophilic compounds such as carboplatin can also be directly affected by obesity.^40^ As adipose tissue consists of relatively more fat than water molecules, hydrophilic drugs such as carboplatin will not easily penetrate adipose tissue. Consequently, it could be assumed that weight descriptors accounting for excess fatty tissue (eg, AIBW) would be more accurate indicators of carboplatin clearance than actual body weight.

Some studies have suggested using CKD-EPI for improved carboplatin dosing.^41,42^ However, in our study, CKD-EPI based on creatinine or creatinine + cystatin C underestimated carboplatin exposure with increasing BMI (see **Supplemental Digital Content 4**, http://links.lww.com/TDM/A834). Moreover, the conventional CG resulted in a better approximation of carboplatin clearance than CKD-EPI creatinine across all BMI groups. Hence, our study suggests, in contrast with the literature, that CKD-EPI does not improve dosing compared with conventional CG.

The sample size (and thus the statistical power) of this study was determined based on the different pharmacokinetic parameters expected in each BMI subgroup. Previous studies showed an average overestimation of carboplatin exposure (AUC) in patients who had obesity 30%–35%, and hence, this study was powered on these observations.^2^ Consequently, based on a maximum difference of AUC of 30% between BMI categories, 7 patients for each BMI subgroup should have been enlisted for sufficient power to make a significant conclusion concerning primary outcomes. Unfortunately, none of the BMI subgroups reached the anticipated number of patients included due to time and resource limitations and the exclusion of patients based on insufficient pharmacokinetic data due to sampling issues. In addition, we initially anticipated based on previous data that at least 1 in 4 patients would have a serum creatinine concentration below 60 µmol/L; however, only 2 such patients (out of 18) were identified. Therefore, it was impossible to perform statistical testing and draw an unambiguous conclusion, which should be considered an important limitation of this study. Moreover, in our study, the differences in carboplatin exposure between BMI categories were not at all near the >30% overestimation as previously reported, indicating that more patients were needed to prove a significant difference.^2^

Advances in deep learning and medical imaging could provide an opportunity for the complete evaluation of body composition and creatinine clearance in patients with cancer. Studies have shown the possibility of using CT scans to acquire body composition estimates such as muscle and fat volumes.^43^ More specifically, the cross-sectional muscle area at the L3 level is strongly associated with total muscle volume, and thus, creatinine excretion.^44,45^ Indeed, recent studies using deep learning body composition analyses of clinically acquired CT scans have shown the possibility of estimating creatinine excretion with high accuracy using the L3 cross-sectional muscle area.^46,47^ Consequently, CT scans can potentially be used to predict creatinine clearance, and thus, carboplatin exposure. In addition, other 100% glomerular filtration biomarkers besides cystatin C are being investigated, including pro-encephalin^48^ and iohexol.^49^ However, assessing GFR based on these biomarkers is often complex, expensive, and time-consuming,^36^ and prospective evaluation is required before implementation in clinical practice.

## CONCLUSIONS

In conclusion, our study did not find a preference for using AIBW, substituting low creatinine concentrations with a value of 60 μmol/L and capping estimated creatinine clearance at a maximum of 125 mL/min for patients with a BMI >25.0 kg/m^2^ over the conventional Cockcroft–Gault formula. However, the biomarker cystatin C using the formula of Schmitt et al^16^ approximated the target AUC almost perfectly in all BMI groups. Hence, our study suggests that using cystatin C should be considered as an improved strategy for safe carboplatin dosing.

## Supplementary Material
