# Supplementary material for: Pharmacokinetic Study of Carboplatin Using Various Overweight-Correcting Dosing Algorithms and Biomarkers in Patients With Varying BMI Categories
Source: Ther Drug Monit. 2025 Apr 29;47(6):730–9. doi: 10.1097/FTD.0000000000001328 (PMC12588668; doi:10.1097/FTD.0000000000001328)
Supplement: Supplementary file 1 [file tdm-47-0730-s001.docx]

**Pharmacokinetic study of carboplatin using various overweight-correcting dosing algorithms and biomarkers in patients with varying BMI categories**

M.P. Kicken, PharmD ^1,2,3^; C. Bethlehem, PharmD^1,4^; K. Beunen, MSc^5^; Y. P. de Jong, MD^6^; T. van Voorthuizen, MD^5^; J.J. van den Hudding, PharmD^1^; D.J.A.R. Moes, PharmD PhD^7^; M. van Luin, PharmD PhD^1,8^; R. ter Heine, PharmD PhD^3^; H.J.M. Smit, MD PhD^6^; P.M.G. Filius, PharmD PhD^1^; M.J. Deenen, PharmD PhD^1,2,7^

^1^ Department of Clinical Pharmacy, Rijnstate Hospital, Arnhem, the Netherlands

^2^ Department of Clinical Pharmacy, Catharina Hospital, Eindhoven, the Netherlands

^3^ Department of Pharmacy, Radboud University Medical Center, Radboud Institute for Health Sciences, Nijmegen, the Netherlands

^4^ Department of Clinical Pharmacy, Erasmus University Medical Center, Rotterdam, the Netherlands

^5^ Department of Internal Medicine, Rijnstate Hospital, Arnhem, the Netherlands

^6^ Department of Pulmonology, Rijnstate Hospital, Arnhem, the Netherlands

^7^ Department of Clinical Pharmacy and Toxicology, Leiden University Medical Center, Leiden, the Netherlands

^8^ Department of Clinical Pharmacy, Meander Medical Center, Amersfoort, the Netherlands

# SUPPLEMENTARY MATERIAL

## Supplementary S1: carboplatin dosing formulas

| **Calvert formula [1]** |
| --- |
| $DOSE [mg]=\mathrm{AUC}_{\mathrm{target}}*(GFR+25)$ |

**International System (SI) units:** AUC in mg/mL*min, GFR in mL/min

| **The Cockcroft Gault formula [2]^1^** |
| --- |
| $estimated CrCL [mL/min]=\frac{\left( 140-AGE \right)*WEIGHT}{0.815*\mathrm{Cr}_{\mathrm{SERUM}}}*0.85 \left[ IF FEMALE \right]$ |

**SI units:** age in years, weight in kg, serum creatinine in mg/dL

| **Schmitt *et al.* cystatin C formula [3]^1^** |
| --- |
| $CL [mL/min]=117.8*\left( \frac{\mathrm{Cr}_{\mathrm{SERUM}}}{75} \right)^{-0.450}*\left( \frac{{cystatin C}_{\mathrm{SERUM}}}{1.0} \right)^{-0.385}*\left( \frac{\mathrm{WEIGHT}}{65} \right)^{+0.504}*\left( \frac{\mathrm{AGE}}{56} \right)^{-0.366}*0.847 [IF FEMALE]$ |

**SI units:** serum creatinine in μmol/L, cystatin C in mg/L, weight in kg, age in years

| **Flat dosing [4, 5]** |
| --- |
| $Flat dosing \left[ \mathrm{mg} \right]=carboplatin population clearance *\mathrm{AUC}_{\mathrm{target}}$ |

**SI units:** carboplatin population clearance in mL/min, AUC in mg/mL*min

| **CKD-EPI creatinine formula [6]^2^** | |
| --- | --- |
| $eGFR [mL/min/1.73m^{2}]=142*\left( \frac{\mathrm{Cr}_{\mathrm{SERUM}}}{A} \right)^{B}*{0.9938}^{\mathrm{AGE}}*1.012 [IF FEMALE]$ | |
| If *female* and:   - Serum creatinine ≤ 0.7: use A = 0.7 and B = -0.241 - Serum creatinine > 0.7: use A = 0.7 and B = -1.200 | If *male* and:   - Serum creatinine ≤ 0.9: use A = 0.9 and B = -0.302 - Serum creatinine > 0.9: use A = 0.9 and B = -1.200 |

**SI units:** serum creatinine in mg/dL, age in years

| **CKD-EPI creatinine + cystatin C formula [6]^2^** | |
| --- | --- |
| $eGFR [mL/min/1.73m^{2}]=135*\left( \frac{\mathrm{Cr}_{\mathrm{SERUM}}}{A} \right)^{B}*\left( \frac{{cystatin C}_{\mathrm{SERUM}}}{C} \right)^{D}*{0.9961}^{\mathrm{AGE}}*0.963 [IF FEMALE]$ | |
| If *female* and:   - Serum creatinine ≤ 0.7 and serum cystatin C ≤ 0.8:   A = 0.7, B = -0.219, C = 0.8, and D = -0.323   - Serum creatinine ≤ 0.7 and serum cystatin C > 0.8:   A = 0.7, B = -0.219, C = 0.8, and D = -0.778   - Serum creatinine > 0.7 and serum cystatin C ≤ 0.8:   A = 0.7, B = -0.544, C = 0.8, and D = -0.323   - Serum creatinine > 0.7 and serum cystatin C > 0.8:   A = 0.7, B = -0.544, C = 0.8, and D = -0.778 | If *male* and:   - Serum creatinine ≤ 0.9 and serum cystatin C ≤ 0.8:   A = 0.9, B = -0.144, C = 0.8, and D = -0.323   - Serum creatinine ≤ 0.9 and serum cystatin C > 0.8:   A = 0.9, B = -0.144, C = 0.8, and D = -0.778   - Serum creatinine > 0.9 and serum cystatin C ≤ 0.8:   A = 0.9, B = -0.544, C = 0.8, and D = -0.323   - Serum creatinine > 0.9 and serum cystatin C > 0.8:   A = 0.9, B = -0.544, C = 0.8, and D = -0.778 |

**SI units:** serum creatinine in mg/dL, cystatin C in mg/L, age in years

^1^ Estimated clearance [mL/min] is used as substitute of the GFR in the Calvert formula

^2^ Estimated clearance [mL/min/1.73m^2^] is adjusted for body surface area (BSA) and then used as substitute of GFR in the Calvert formula

## Supplementary S2: Weight descriptors used in the Cockcroft-Gault formula

| **Weight descriptor*** | **Equation** |
| --- | --- |
| Ideal Body Weight (IBW) | IBW [kg] = 49.9 + 0.89 x (HEIGHT [cm] - 152.4) for men  IBW [kg] = 45.4 + 0.89 x (HEIGHT [cm] - 152.4) for women |
| Adjusted Ideal Body Weight (AIBW) | AIBW [kg] = IBW + 0.4 x (ABW – IBW) |
| Bénézet formula [7] | Bénézet [kg] = (IBW + ABW) x 0.512 |

**Abbreviations:** ABW = absolute body weight

*All weight descriptors have the SI unit [kg]

## REFERENCES

1. Calvert AH, Newell DR, Gumbrell LA, O'Reilly S, Burnell M, Boxall FE, et al. Carboplatin dosage: prospective evaluation of a simple formula based on renal function. J Clin Oncol. 1989;7(11):1748-56.

2. Cockcroft DW, Gault MH. Prediction of creatinine clearance from serum creatinine. Nephron. 1976;16(1):31-41.

3. Schmitt A, Gladieff L, Lansiaux A, Bobin-Dubigeon C, Etienne-Grimaldi MC, Boisdron-Celle M, et al. A universal formula based on cystatin C to perform individual dosing of carboplatin in normal weight, underweight, and obese patients. Clin Cancer Res. 2009;15(10):3633-9.

4. Ekhart C, de Jonge ME, Huitema AD, Schellens JH, Rodenhuis S, Beijnen JH. Flat dosing of carboplatin is justified in adult patients with normal renal function. Clin Cancer Res. 2006;12(21):6502-8.

5. Ekhart C, Rodenhuis S, Schellens JH, Beijnen JH, Huitema AD. Carboplatin dosing in overweight and obese patients with normal renal function, does weight matter? Cancer Chemother Pharmacol. 2009;64(1):115-22.

6. Inker LA, Eneanya ND, Coresh J, Tighiouart H, Wang D, Sang Y, et al. New Creatinine- and Cystatin C-Based Equations to Estimate GFR without Race. N Engl J Med. 2021;385(19):1737-49.

7. Bénézet S, Guimbaud R, Chatelut E, Chevreau C, Bugat R, Canal P. How to predict carboplatin clearance from standard morphological and biological characteristics in obese patients. Ann Oncol. 1997;8(6):607-9.

8. Akgül S, Chan BA, Manders PM. Carboplatin dose calculations for patients with lung cancer: significant dose differences found depending on dosing equation choice. BMC Cancer. 2022;22(1):829.

## Supplementary S3: different concentration curves of each patient

The pink dots represent measured carboplatin concentrations over time, while the blue dots represent predicted carboplatin concentrations over time based on the NONMEM model by Ekhart *et al.* [4, 5].

| **PATIENT 1 (BMI < 25 kg/m^2^): AUC = 5.57 mg/mL*min** |
| --- |
| **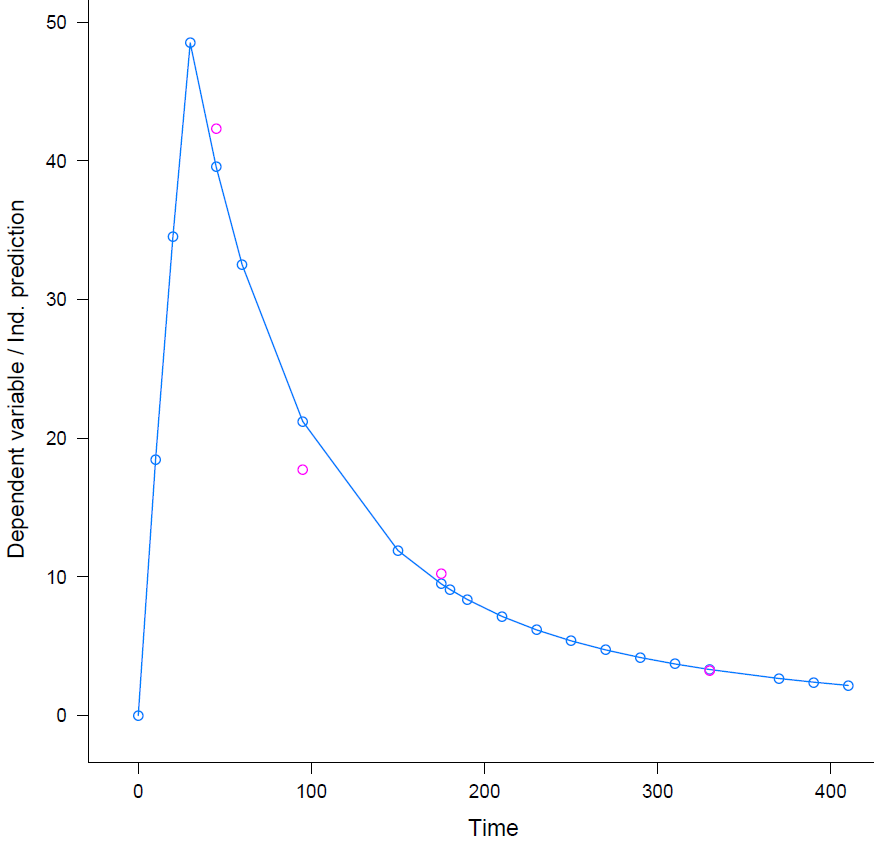** |

| **PATIENT 2 (BMI < 25 kg/m^2^): AUC = 5.49 mg/mL*min** |
| --- |
| **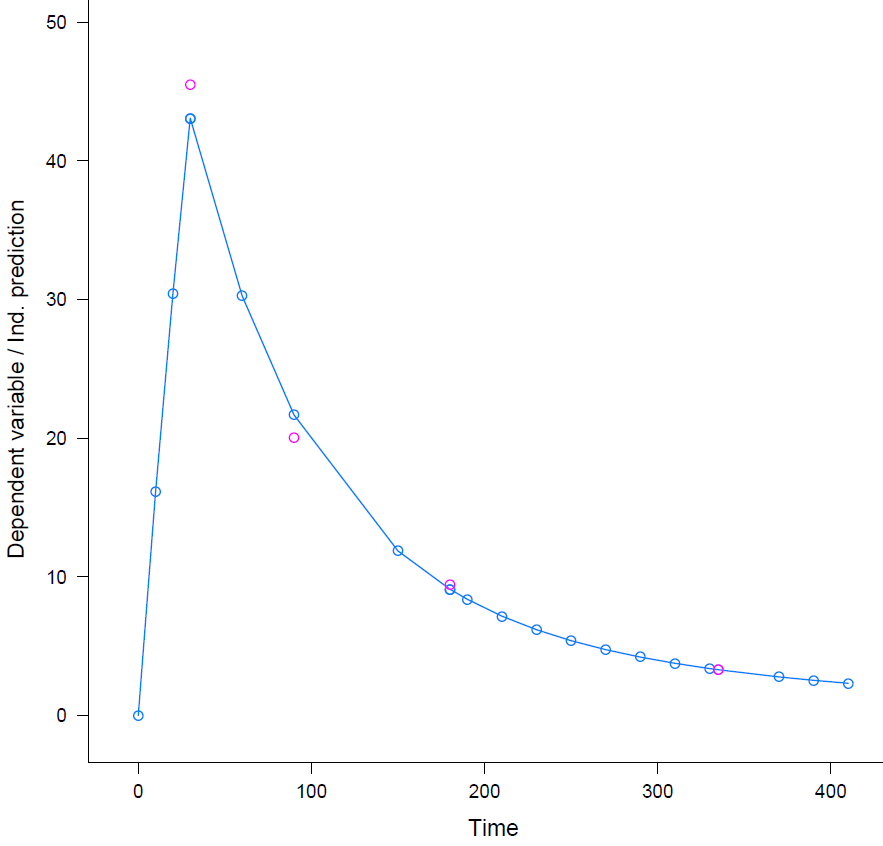** |
| **PATIENT 3 (BMI 25 – 30 kg/m^2^): AUC = 5.35 mg/mL*min** |
| **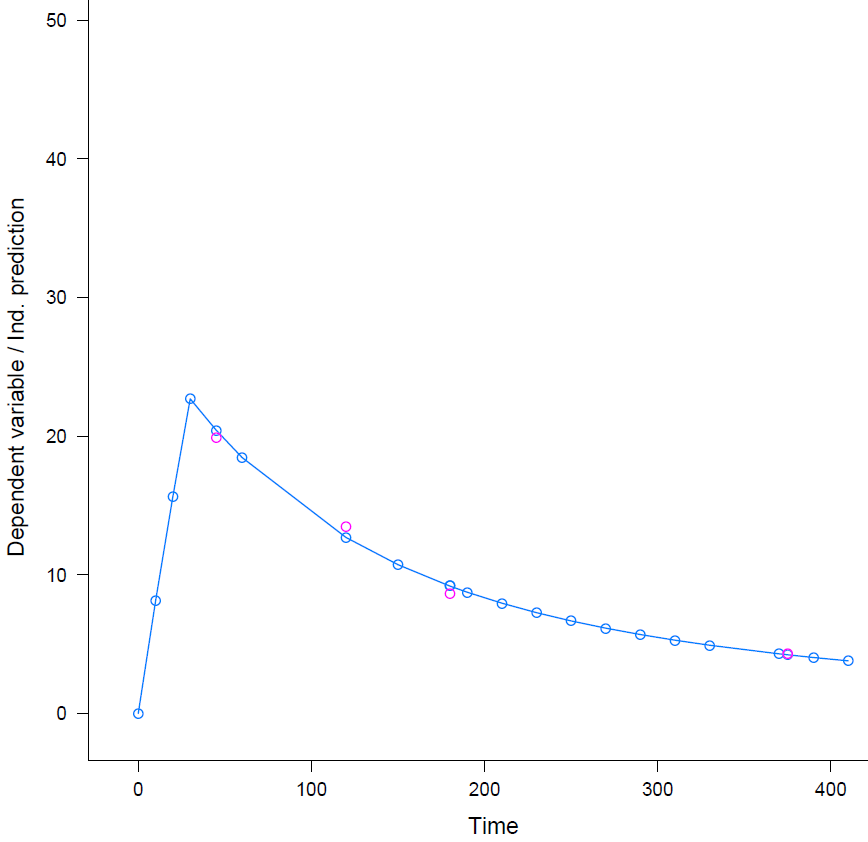** |

| **PATIENT 4 (BMI ≥ 30 kg/m^2^): AUC = 5.54 mg/mL*min** |
| --- |
| **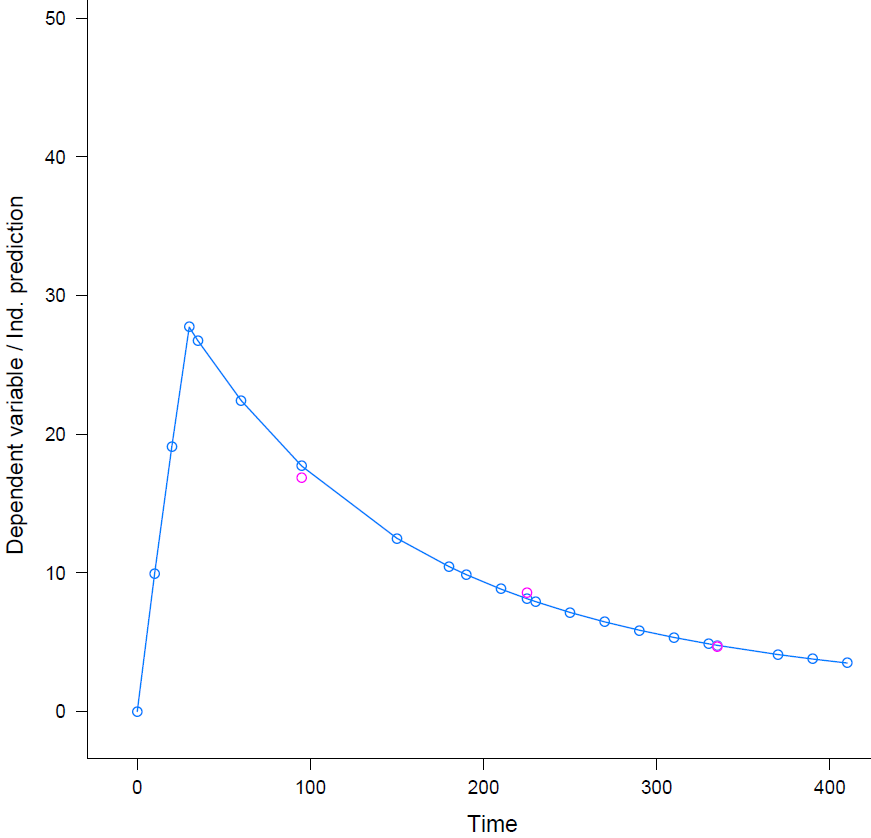** |
| **PATIENT 5 (BMI < 25 kg/m^2^): AUC = 6.49 mg/mL*min** |
| **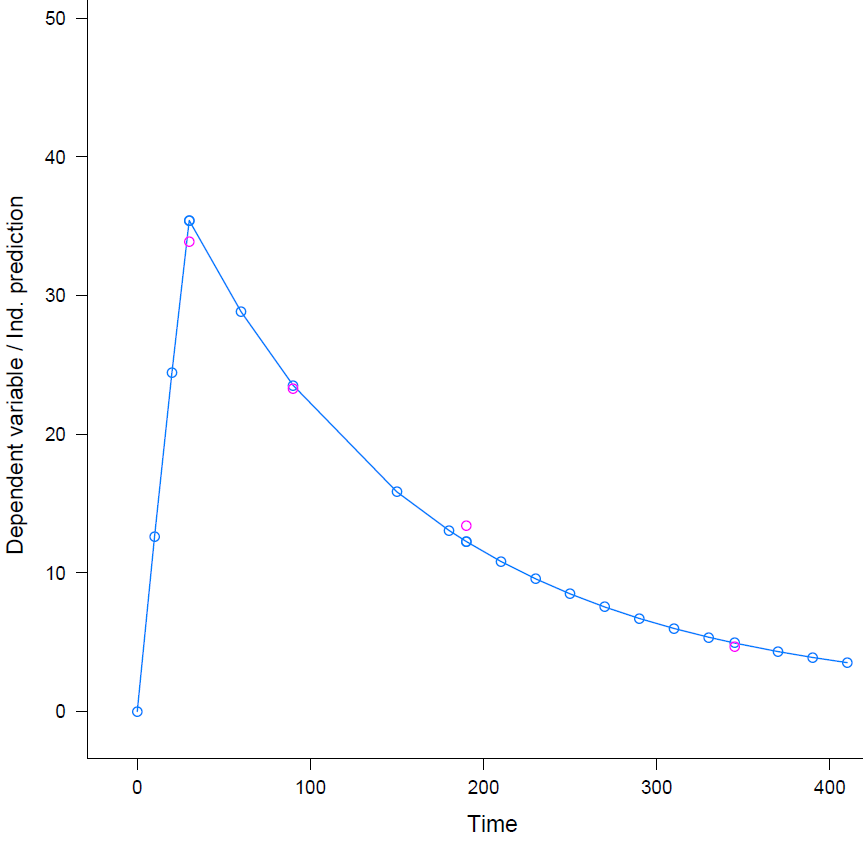** |

| **PATIENT 6 (BMI < 25 kg/m^2^): AUC = 5.72 mg/mL*min** |
| --- |
| **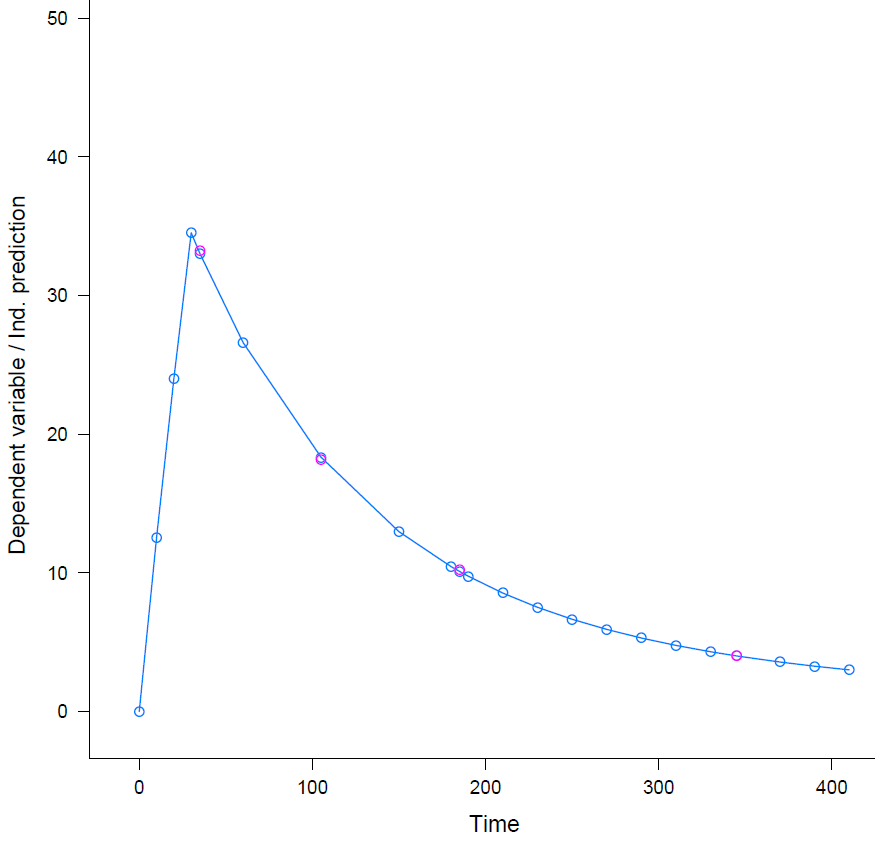** |
| **PATIENT 7 (BMI 25 – 30 kg/m^2^): AUC = 5.84 mg/mL*min** |
| **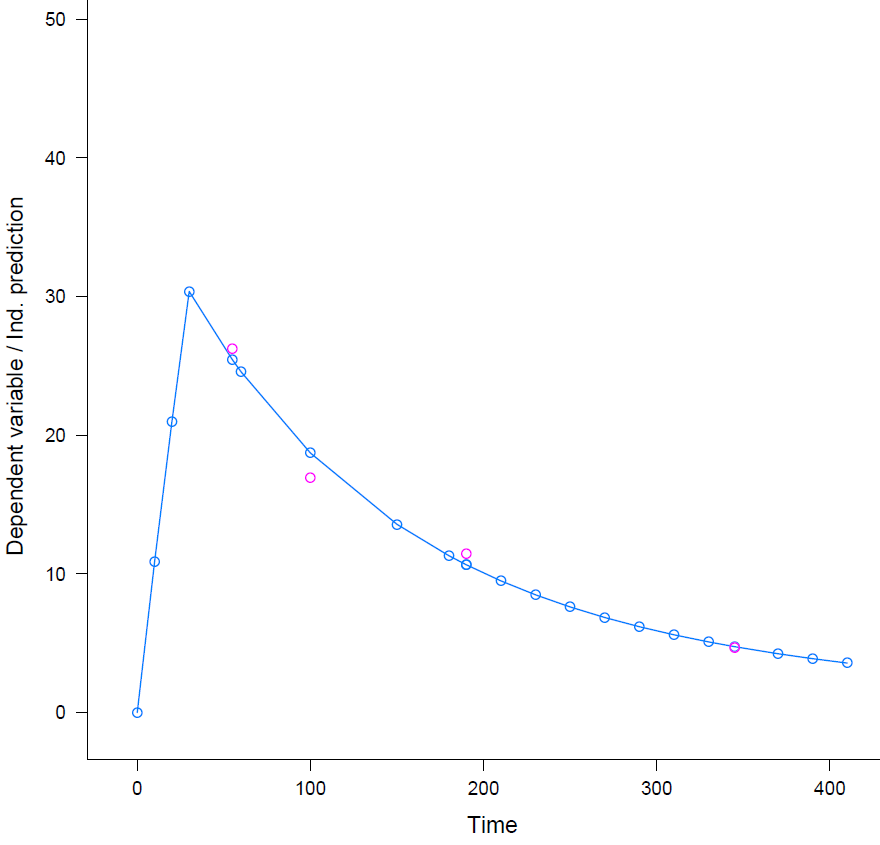** |

| **PATIENT 8 (BMI < 25 kg/m^2^): AUC = 4.81 mg/mL*min** |
| --- |
| **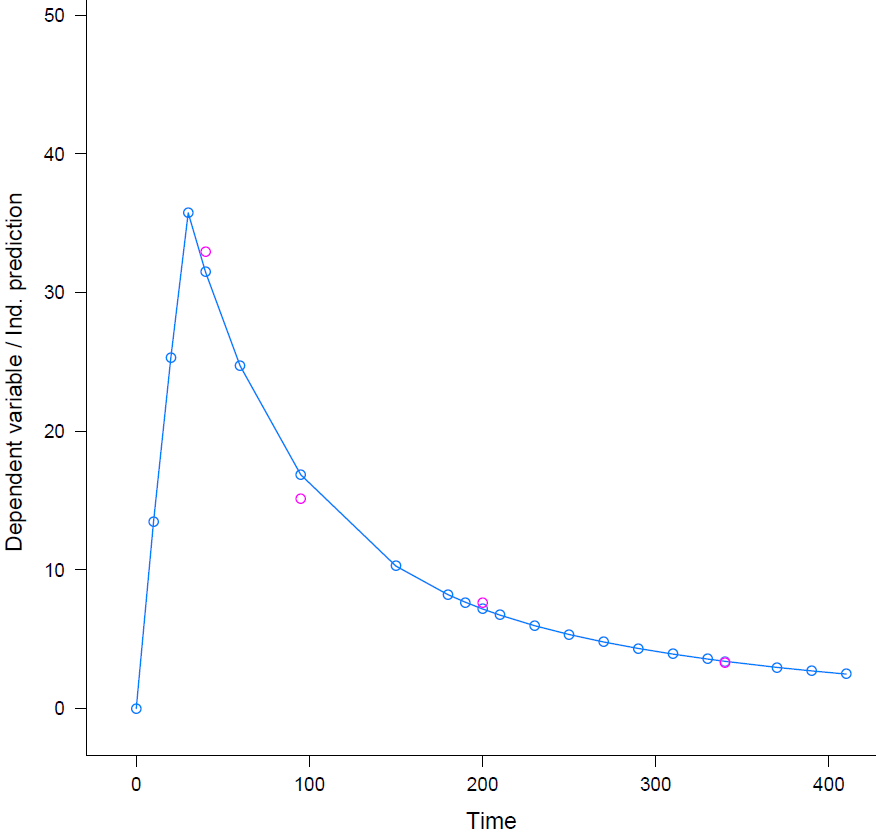** |
| **PATIENT 9 (BMI < 25 kg/m^2^): AUC = 5.29 mg/mL*min** |
| **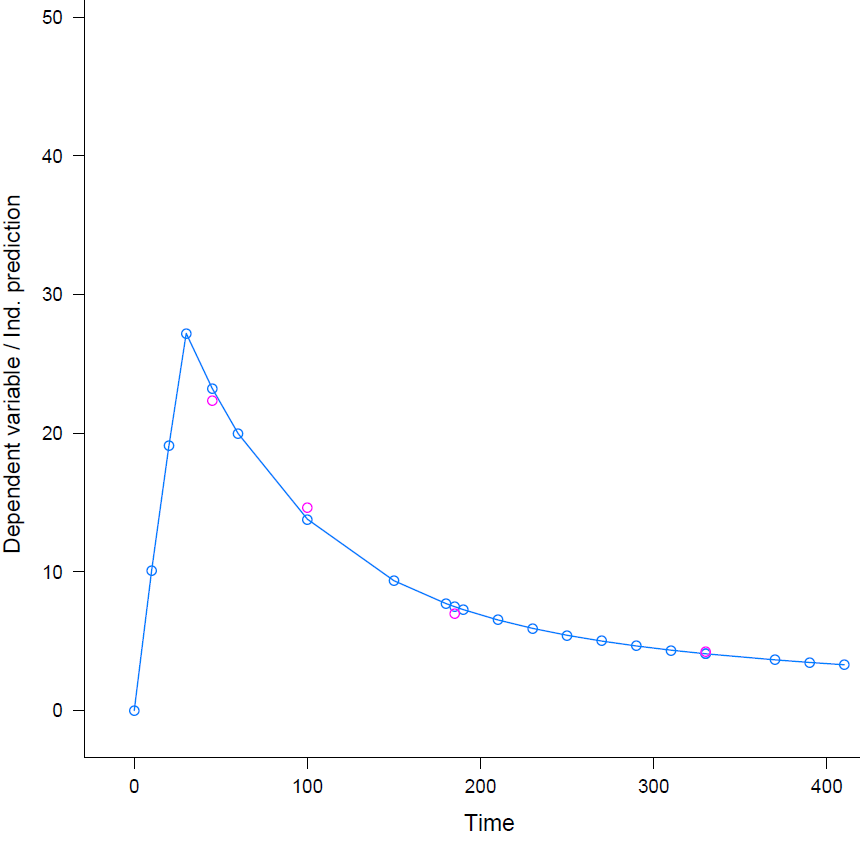** |

| **PATIENT 10 (BMI < 25 kg/m^2^): AUC = 5.77 mg/mL*min** |
| --- |
| **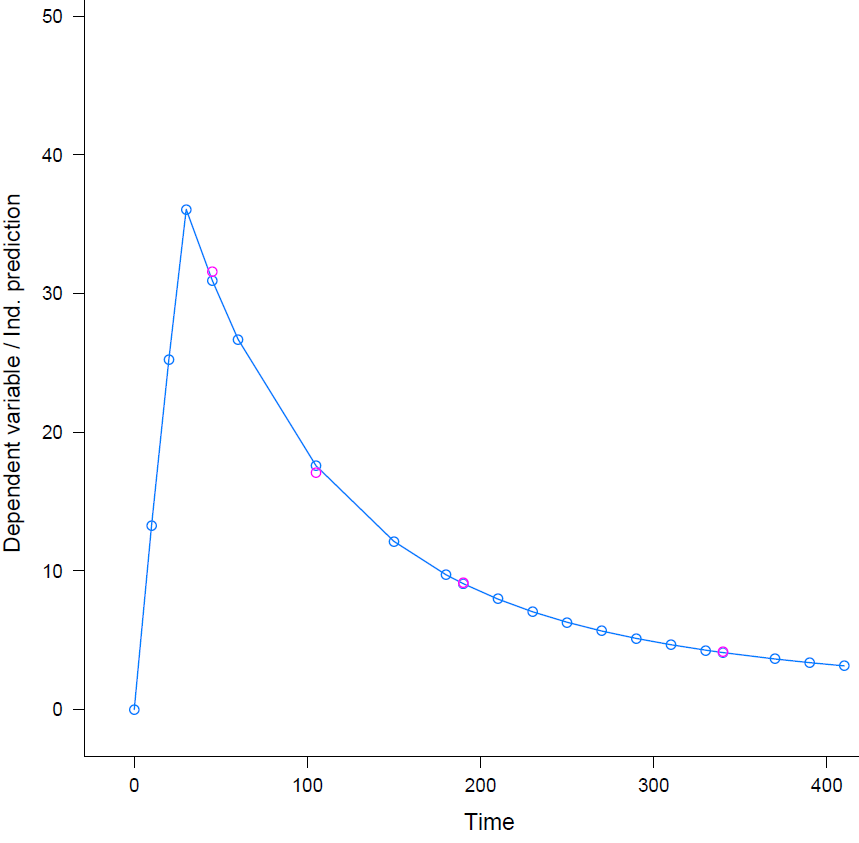** |
| **PATIENT 11 (BMI ≥ 30 kg/m^2^): AUC = 4.61 mg/mL*min** |
| **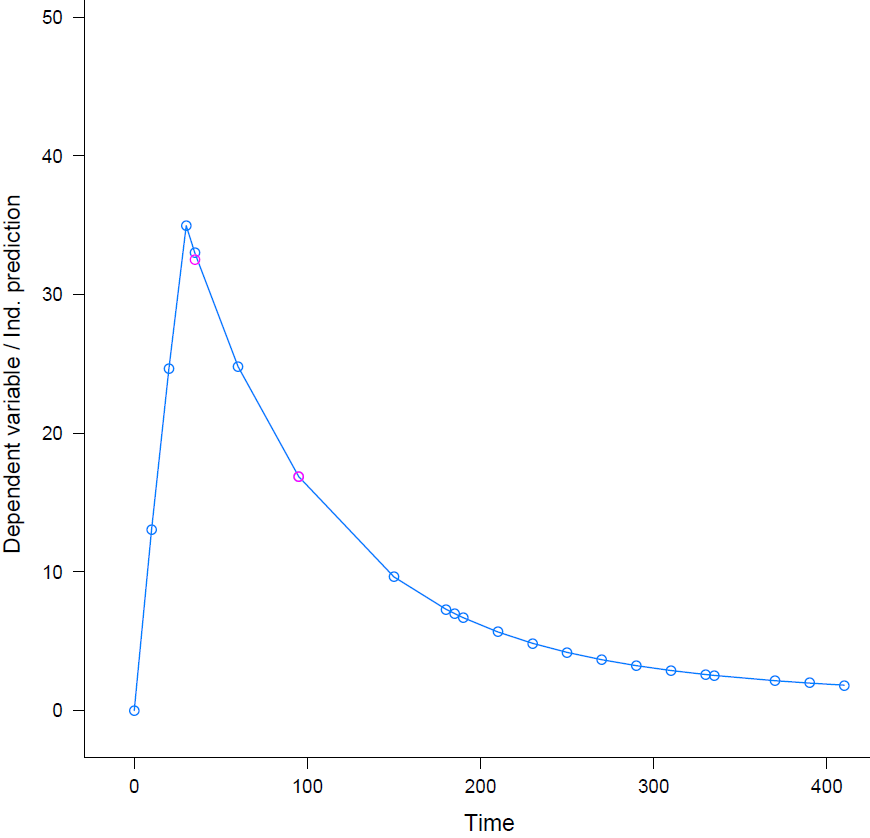** |

| **PATIENT 12 (BMI 25 – 30 kg/m^2^): AUC = 5.84 mg/mL*min** |
| --- |
| **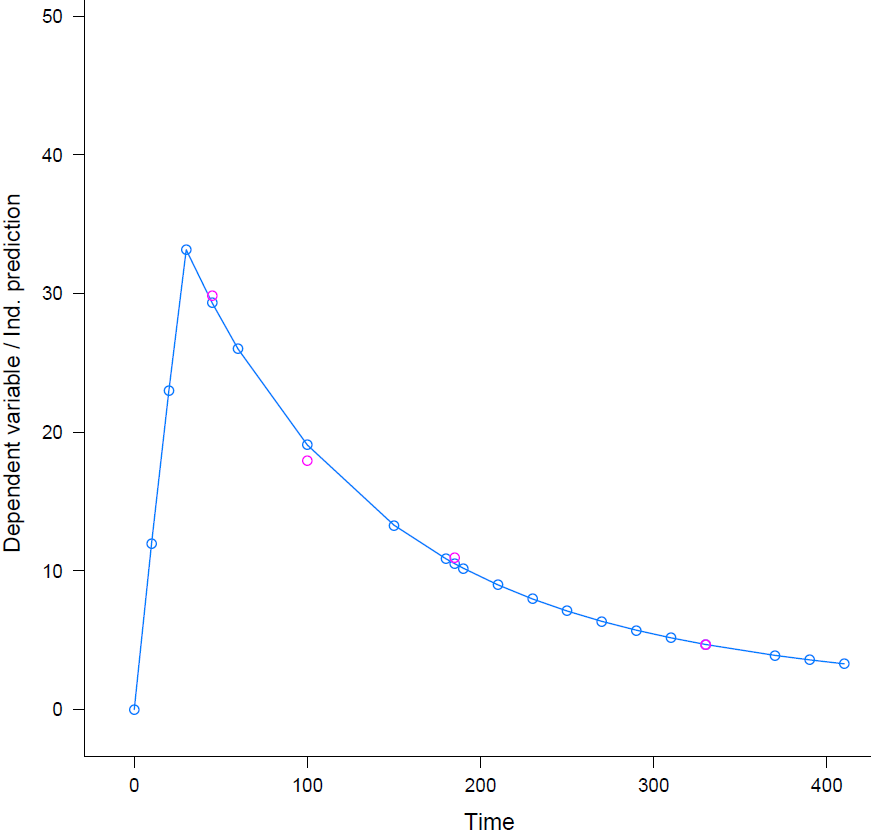** |
| **PATIENT 13 (BMI ≥ 30 kg/m^2^): AUC = 6.10 mg/mL*min** |
| **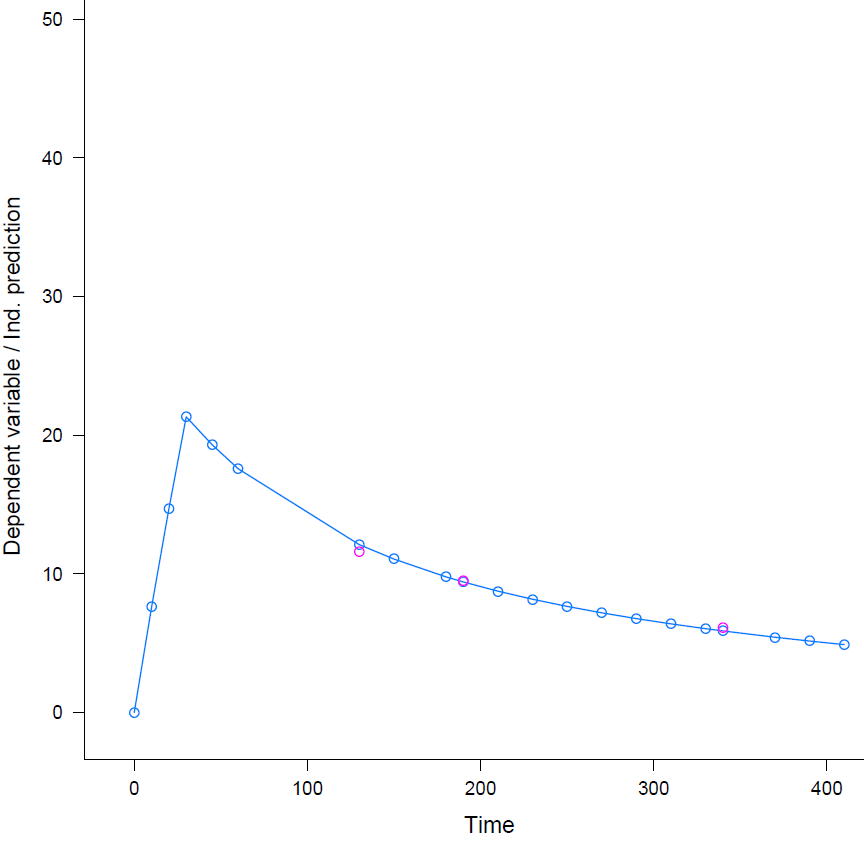** |

| **PATIENT 14 (BMI 25 – 30 kg/m^2^): AUC = 5.84 mg/mL*min** |
| --- |
| **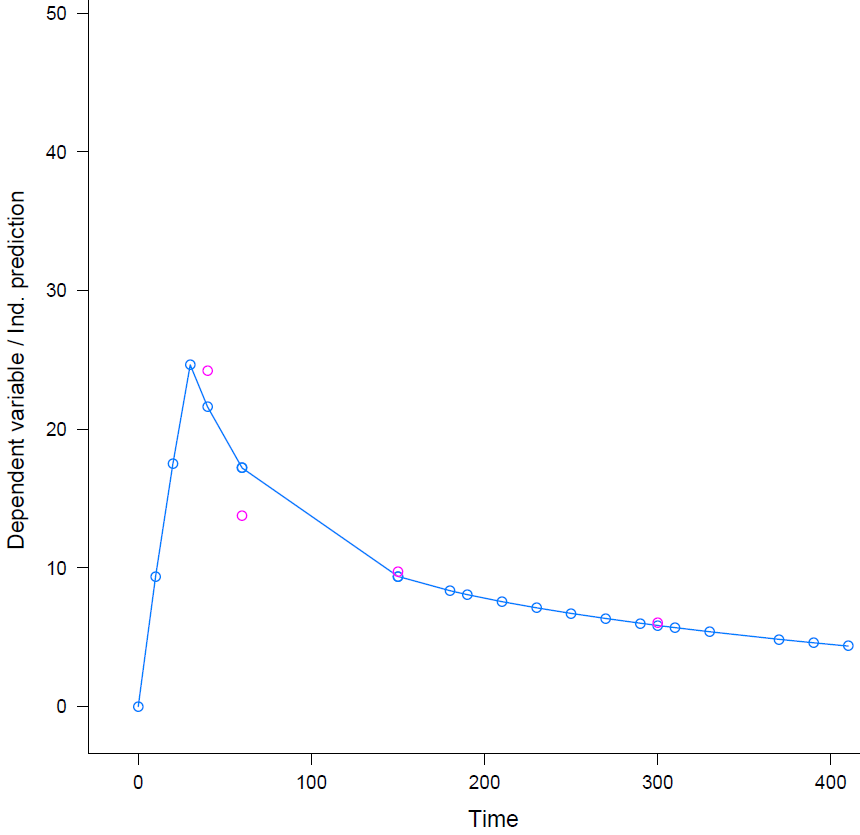** |

| **PATIENT 15 (BMI ≥ 30 kg/m^2^): AUC = 6.00 mg/mL*min** |
| --- |
| **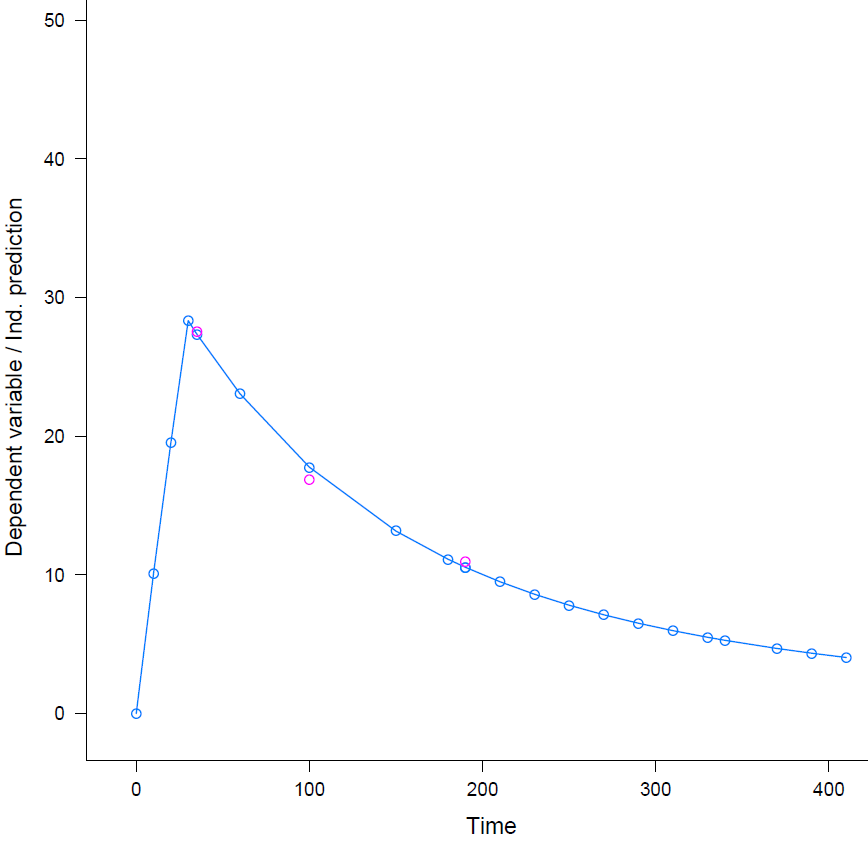** |

| **PATIENT 16 (BMI ≥ 30 kg/m^2^): AUC = 4.39 mg/mL*min** |
| --- |
| **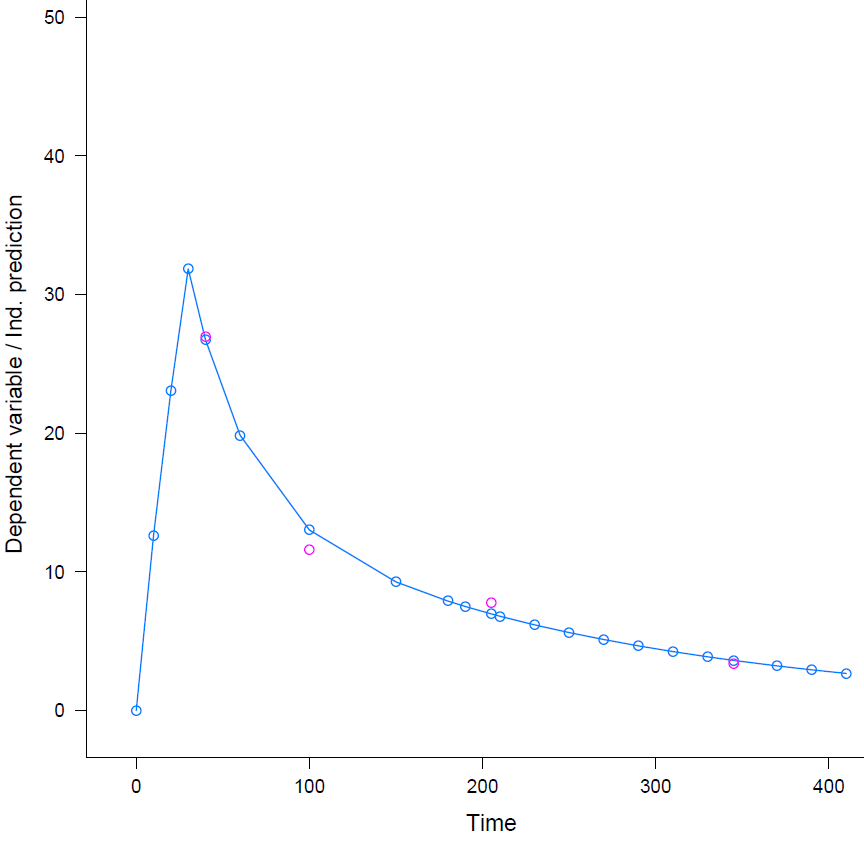** |
| **PATIENT 17 (BMI ≥ 30 KG/M^2^): AUC = 4.10 mg/mL*min** |
| **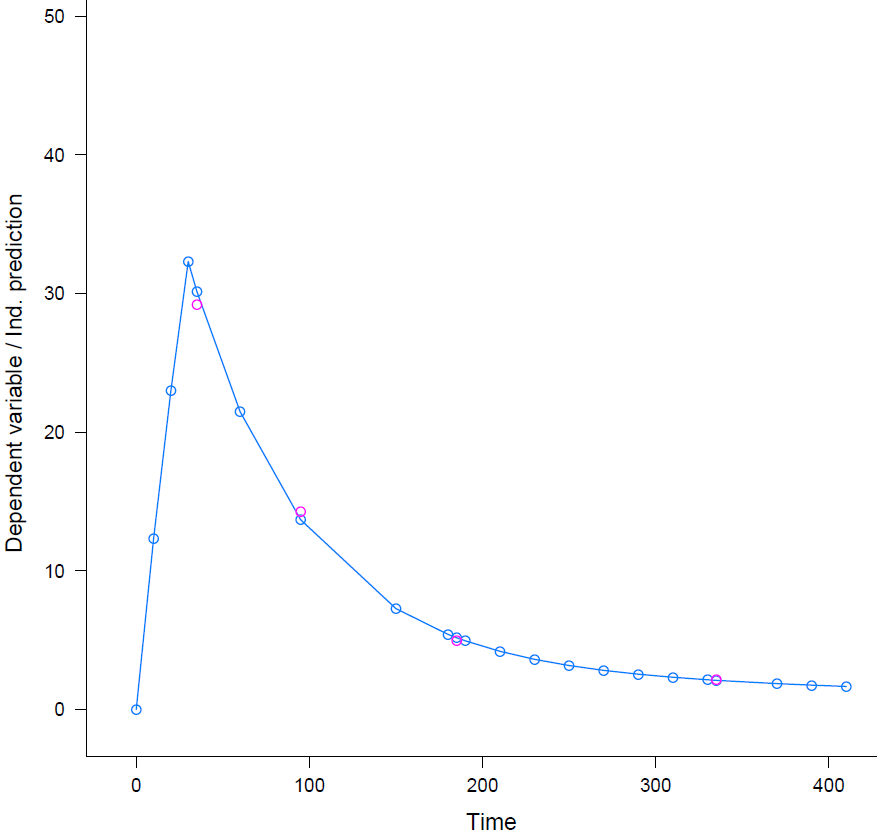** |

| **PATIENT 18 (BMI 25 – 30 KG/M^2^): AUC = 5.84 mg/mL*min** |
| --- |
| **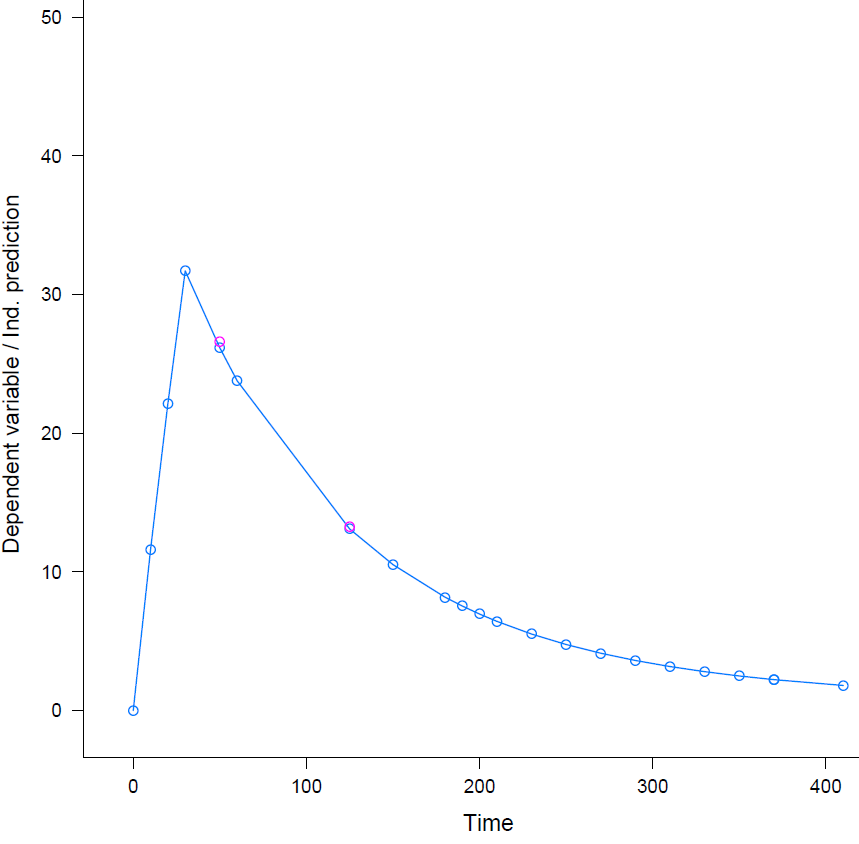** |

## Supplementary S4: AUC (expressed as MPE%) of different estimators of GFR relative to target AUC


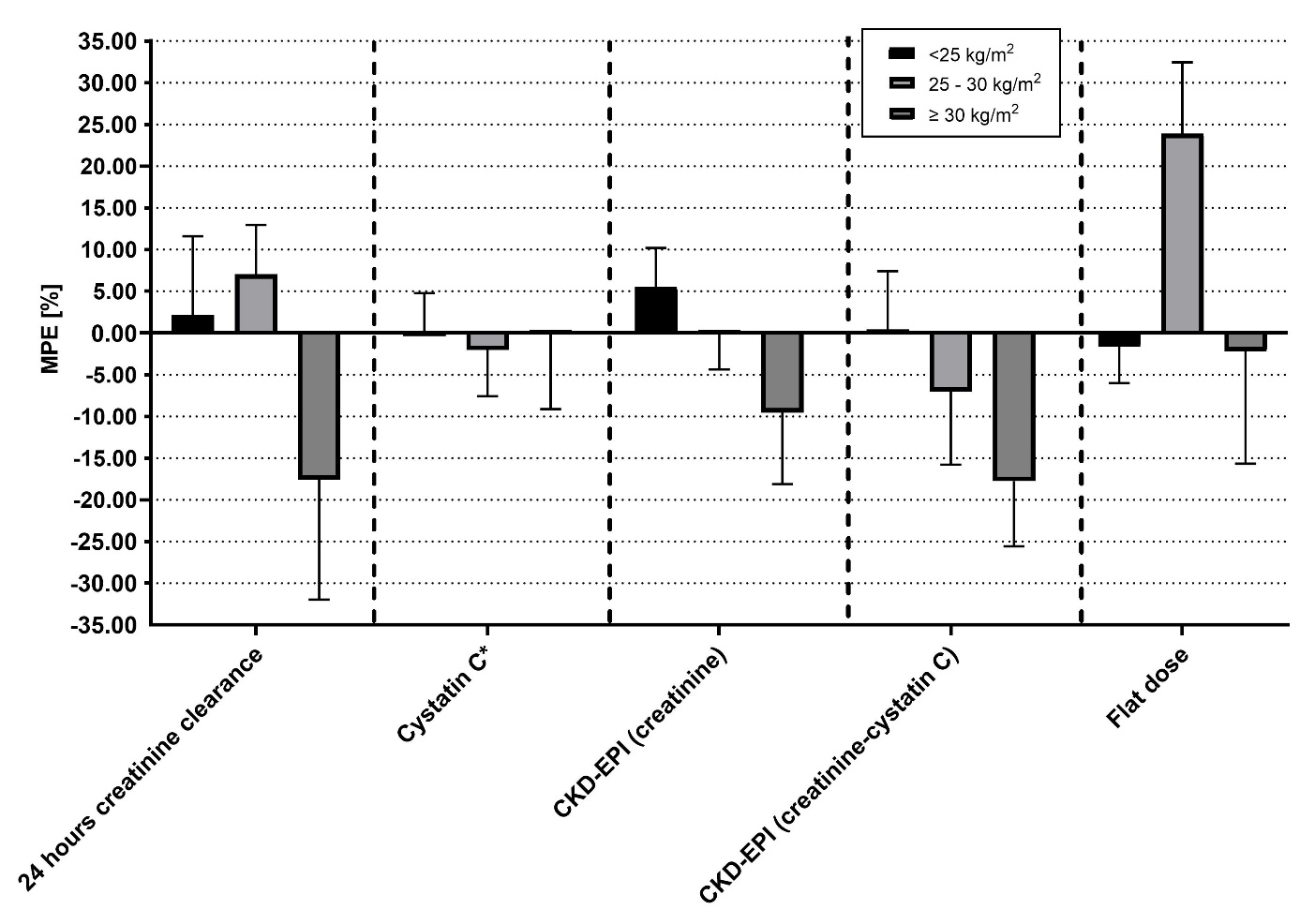


* Following the cystatin C formula of Schmitt *et al.* [3]

**Abbreviations**: AUC = area under the curve, MPE = mean percentage error, GFR = glomerular filtration rate, CKD-EPI = Chronic Kidney Disease Epidemiology Collaboration. Margins of error are equal to the standard error (SE) of the mean of the particular GFR estimator. The mean carboplatin population clearance for flat dosing was 112.4 mL/min.
